# Beyond Compliance: Understanding the Role of Peer Review Through a Theory of Change

**DOI:** 10.1111/jep.70422

**Published:** 2026-03-26

**Authors:** Axel Kaehne, Julie Feather, Tom Simcock

**Affiliations:** ^1^ Medical School Edge Hill University Ormskirk UK; ^2^ Evaluation and Policy Analysis Unit Edge Hill University Ormskirk UK; ^3^ School of Human and Health Sciences University of Huddersfield Ormskirk UK

## Abstract

**Objective:**

Peer review is a widely used mechanism to support quality improvement in healthcare, yet its effectiveness remains uncertain. This study aims to examine how peer review visits contribute to care quality improvement in NHS England's specialised commissioned services, and to develop a theory of change that explains their mechanisms of action.

**Methods:**

A theory‐based evaluation was undertaken using qualitative methods. Data were collected in two phases: (1) 17 semi‐structured interviews with peer reviewers, NHS staff, commissioners, and patient representatives; and (2) two stakeholder workshops with directors of nursing and service commissioners. Thematic analysis using deductive coding was guided by the Impact Domain Framework. Findings were synthesised into a logic model and Theory of Change, visualised using Directed Acyclic Graphs (DAGs), incorporating relational dimensions of peer review.

**Results:**

Analysis of transcripts from a diverse range of stakeholders produced four core themes: the tension between compliance and learning objectives; the role of trust and relational dynamics; limited mechanisms for knowledge exchange; and a lack of follow‐up impeding sustained improvement. The final Theory of Change model distinguishes between compliance‐driven and learning‐driven pathways, integrating relational factors such as trust and engagement as key enablers of improvement.

**Conclusion:**

Peer review visits are more effective when perceived as opportunities for learning rather than compliance monitoring. The study offers a novel Theory of Change and visual model to guide future evaluation and practice, emphasising the importance of relational dynamics and sustained follow‐up. These insights should inform both implementation and policy by supporting a shift toward trust‐based, developmental peer review models that strengthen organisational learning and align with the goals of a learning health system.

## 1 Introduction

Peer review has long been recognised as a mechanism for ensuring and improving healthcare quality internationally [1, 2]. It is widely used in hospital accreditation, clinical audits, and medical professional revalidation to assess compliance with quality standards and drive improvements. However, its effectiveness varies significantly, with studies highlighting inconsistencies in benchmarking criteria, implementation approaches, and outcomes [3]. In particular, peer review remains under‐theorised in healthcare, with limited empirical studies explaining which mechanisms lead to success or failure.

While some research suggests peer reviews lead to measurable quality improvements [4], other studies question their impact, reporting mixed evidence and a lack of cost‐effectiveness data (C.M. Roberts et al. [5]; H. Roberts, D. Van Der Winden [6, 7]). These uncertainties make it essential to better understand how peer review mechanisms function, which aspects drive change, and which factors influence their clinical effectiveness.

Peer review has traditionally been viewed through two distinct lenses. Firstly, it is often perceived to be a quality control mechanism, assessing adherence to professional and regulatory standards (U. Nimptsch [8]). Secondly, it can also be seen as a way to support quality improvement, driving accountability, learning, and improved patient outcomes [2]. There is some evidence to support the latter purpose. A study within the Californian prison healthcare system found that structured peer review was associated with significant reductions in mortality rates, reinforcing its role in improving clinical outcomes [2].

While peer review is well established in nurse education, business, and publishing [9, 10], its application in healthcare is more complex. Studies have identified challenges related to professional hierarchies, cognitive biases, and the reluctance of clinicians to critique peers (Dignum et al. [11], George et al. [12]).

Some regulatory frameworks also shape the effectiveness of peer reviews. For example, in some jurisdictions, legal protections under healthcare quality improvement legislation influence the conditions under which peer reviews occur [13]. Similarly, incorporating patient perspectives into peer review structures may enhance transparency but shift the emphasis from professional reflection to external accountability (Braun et al. [14]).

The literature on peer review spans multiple domains. Studies indicate benefits for professional development and patient safety but provide limited evidence of clinical impact [15, 16, 17]. External reviews of healthcare organisations also often produce insights but studies have demonstrated that they lead to only limited systemic change [7, 18, 19]. Finally, practice visits and structured peer review models have come to the attention of researchers and evaluators who were able to demonstrate some yet inconsistent improvements in treatment adherence and care quality [20, 21].

Some studies suggest peer review enhances adherence to guidelines and reduces performance variability in clinical settings, such as cardiac surgery [22, 23, 24], Veterans Affairs hospitals [25], and chronic disease management (Parsons et al. [26]). However, a lack of structured evaluation frameworks and causal models makes it difficult to determine why peer review works in some contexts but not in others.

The lack of clarity in peer review mechanisms presents challenges for clinicians and healthcare organisations seeking to improve service quality. Studies suggest that peer review may be more effective when embedded into routine clinical meetings or structured learning environments rather than applied as a top‐down compliance tool [27]. However, inconsistencies in follow‐up mechanisms and the absence of a unified theoretical framework make it difficult to assess which models of peer review are most effective in improving patient care [18, 20, 28].

Although peer review is widely accepted as a tool for healthcare quality assurance, its impact is shaped by how it is structured, implemented, and followed up in clinical settings, echoing complex interventions which are challenging to evaluate with conventional approaches [29, 30]. The balance between compliance‐driven accountability and learning‐based reflection remains critical.

Given the global reliance on peer review as a clinical quality improvement mechanism, a better understanding of its active components is needed. In particular, it appears essential to develop evaluation models, such as a Theory of Change to assess peer review effectiveness. This study contributes to the theoretical and empirical understanding of peer review mechanisms, offering insights that are relevant for healthcare services worldwide by developing a Theory of Change model which can be used in future evaluative studies.

## 2 QNT Led Peer Review Visits in England's NHS

The Quality and Nursing Team (QNT) situated within the National Health Service (NHS) in England monitors the quality and safety of Specialised Commissioned Services, complementing the work of the Clinical Quality Committee. The service was previously called Quality Surveillance Team (QST). Originally focused on cancer services directly commissioned by NHS England, the QNT's remit has evolved alongside changes in commissioning structures. Despite these shifts, NHS England has maintained direct oversight of certain services, with QNT playing a crucial role in quality assurance and improvement.

QNT assesses provider performance using defined quality indicators and conducts peer review visits to support service compliance with national standards. Its core activities include data collection, performance analysis, and risk assessment through the Quality Surveillance Information System (QSIS). The three‐tier surveillance approach consists of routine monitoring, targeted intervention when concerns arise, and peer review visits for in‐depth evaluation.

Peer review visits are conducted by trained multidisciplinary teams who assess services through interviews, document reviews, and site observations. After each visit, an initial report is shared with the service, providing an opportunity to respond before receiving a final version. Services are expected to develop action plans detailing how they will implement recommendations to enhance care quality.

The QNT's peer review model serves both compliance and quality improvement functions. While ensuring adherence to standards, it also identifies areas of good practice and shared learning opportunities. However, some services perceive peer reviews primarily as regulatory exercises rather than as a mechanism for collaborative learning. This tension between compliance and improvement is a recognised challenge in peer review processes.

The limited time available for peer review visits can further restrict engagement, reducing opportunities for meaningful relationship‐building between teams. A lack of follow‐up mechanisms also presents a barrier to sustaining improvements, as peer review recommendations are not widely shared, limiting cross‐organisational learning. Additionally, the extent to which patients should be involved in the process remains a topic of debate, with some arguing that patient participation enhances transparency, while others caution against shifting the focus away from professional peer assessment [31, 32].

At the outset of this study, there was limited empirical evidence on how peer reviews drive quality improvements in specialised commissioned services. It was unclear which mechanisms underpin their effectiveness and how peer review visits might fail to produce meaningful change. The tension between compliance and learning was seen as a fundamental barrier, with staff often viewing visits as regulatory inspections rather than collaborative learning opportunities.

A key gap in understanding was the absence of a clear theoretical model explaining how peer review visits influence service improvement. Without a structured framework, it was difficult to determine how on‐site dynamics contribute to trust, confidence, and cross‐organisational learning, all of which are critical for an effective peer review process.

This project aimed to evaluate the effectiveness of QNT's peer review process in improving care quality for specialised commissioned services. Using a Theory of Change (ToC) approach, we developed a structured model to identify key components of effective peer reviews and map relationships between them. This approach not only reflected QNT's goal of driving positive change but also allowed us to pinpoint strengths and weaknesses in current peer review practices.

We drew on recent research emphasising the relational nature of peer reviews. While traditional frameworks assume a direct link between review visits and service improvement, Etienne [33] argues that effectiveness depends on trust, relational signalling, and how services perceive the intervention. These insights informed our Theory of Change (ToC), guiding our focus on how relational dynamics influence learning and compliance responses. Our ToC model specifically incorporates the role of trust and interaction in shaping impactful peer review processes.

## 3 Methods

We conducted an evaluation study using a theory‐based approach to assess the effectiveness of QNT peer reviews. This paper reports on the development of a Theory of Change (ToC) model, designed to understand how peer reviews drive quality improvement. We initially mapped a logic model of the peer review process before refining and validating a ToC through qualitative research and framework‐based analysis using Smithson and colleagues' impact framework [34]. A theory‐based evaluation approach was selected to enable systematic exploration of causal mechanisms and contextual conditions underlying peer review processes, offering a structured framework that goes beyond description to support explanatory insight which has an advantage over purely descriptive qualitative designs.

Our evaluation question was: How do peer reviews improve care quality in NHS England's specialised commissioned services?

### 3.1 Sampling and Data Collection

We used purposive sampling to capture diverse perspectives, working with QNT, NHS staff, service managers, commissioners, peer reviewers, and patient representatives. Recruitment was facilitated by NHS QNT following agreed criteria. Purposive sampling was used to ensure diversity of perspectives across stakeholder roles involved in the peer review process, including frontline staff, service managers, commissioners, and patient representatives. While participants were recruited via NHS QNT, care was taken to include individuals from both reviewing and reviewed services to mitigate bias and support a balanced understanding of the process.

In Phase 1, we analysed QNT policies, standard operating procedures, and peer review reports, mapping findings against the Impact Domain Framework (Smithson et al. 2019). We then conducted 17 semi‐structured interviews via Microsoft Teams (20–45 min), exploring stakeholder experiences, compliance pressures, and learning dynamics in peer reviews. Table 1.

**Table 1:** Respondents by role in peer review visit.

Role	Number of participants
NHS QNT staff	4
Frontline staff (visited service)	5
Service manager (visited service)	4
Service commissioners and stakeholders	3
Patient Peer Reviewer	1

In phase 2, we conducted two online stakeholder workshops with directors of nursing and service commissioners, the primary beneficiaries of QNT peer reviews. These workshops aimed to validate the logic model and refine our Theory of Change (ToC) based on insights from Phase 1 interviews. Using a collaborative discussion format, we ensured diverse perspectives were considered. Sessions lasted 1.5 to 2 h, were held via Microsoft Teams, and were recorded, transcribed, and pseudonymised for confidentiality.

### 3.2 Ethical Considerations

This service evaluation was conducted in accordance with the Health Research Authority (HRA) in the United Kingdom (UK) guidelines and, as a service evaluation, did not require formal ethical approval. However, participants were informed about the purpose of the evaluation, and consent was obtained verbally before data collection commenced. All data were anonymised before analysis, stored securely, and handled in compliance with the General Data Protection Regulation (GDPR) in the UK to ensure confidentiality. Internally, the ethics committee of Edge Hill University deemed this study a service evaluation not requiring ethical review and approval.

### 3.3 Data Analysis

We applied Braun and Clarke's thematic analysis [35, 36] to interview and workshop transcripts. All data were transcribed verbatim and reviewed for familiarisation. Initial deductive codes were derived from the Impact Domain Framework, which provided predefined domains (e.g., relational, process, structural impacts) to guide the coding structure and ensure alignment with the study's evaluative focus. Two researchers independently coded a subset of transcripts and discussed discrepancies to reach consensus, thereby enhancing analytic rigour. Coding frameworks were then applied across all transcripts, with iterative adjustments made through team discussions. Themes were developed through constant comparison across interviews and workshops and subsequently mapped against the framework to support coherence and explanatory depth. An example of key verbatim quotes is provided in the appendix. Interview and workshop data were analysed concurrently, with cross‐source triangulation used to refine and validate emerging themes.

Thematic findings derived from empirical data directly informed the construction of the logic model by identifying the core mechanisms, contextual conditions, and intermediate outcomes observed across stakeholder accounts. These model components were then sequenced and synthesised into a Theory of Change that reflects how peer review visits are expected to generate learning and improvement, based on the lived experiences and perceptions of participants.

### 3.4 Theory of Change and DAG Development

To construct our Theory of Change model, we utilised AI‐assisted analysis, ensuring alignment with Directed Acyclic Graphs (DAGs) which is a novel approach to visualising causal relationships in peer review processes [37, 38, 39]. This method allowed us to go beyond a simple logic model, providing a sophisticated representation of peer critique, relational learning, and service improvement.

A Theory of Change (ToC) is a structured framework that outlines how and why a particular intervention is expected to bring about change [40, 41, 42]. It identifies the key components of a programme — such as inputs, activities, outputs, outcomes, and impact — and maps out the causal pathways that link these elements together. Unlike a simple logic model, a ToC explicitly accounts for underlying assumptions, external contextual factors, and potential feedback loops that influence the intervention's effectiveness. By articulating these mechanisms, a ToC helps to clarify the conditions necessary for success and supports a more nuanced understanding of how improvements occur. We used the development of a ToC as a preliminary step towards understanding causal pathways in the peer review visit programme. This then allowed us to develop a directed acyclic graph (DAG). All items listed in our logic model informed our discussion about the Theory of Change model. The purpose of developing a Theory of Change was to clarify how peer review drives learning based improvements. We also wanted to integrated relational signalling and knowledge sharing as essential components of bringing about change as evidenced by the work of Etienne [33]. We used a Directed Acyclic Graph to illustrate the developed Theory of Change model.

A Directed Acyclic Graph (DAG) is a visual representation of causal relationships between variables within a system. It was developed by various teams but popularised by Judea Pearl in his seminal work titled ‘The Book of Why’ [39]. DAGs consist of nodes (representing key elements such as inputs, activities, outputs, and outcomes) and arrows (representing the direction of influence or causality between these elements). Unlike traditional linear models, a DAG explicitly illustrates the causal pathways, feedback loops, and contextual influences that shape an intervention's effectiveness. In our case, the construction of the Theory of Change was grounded in empirical themes and supported by the use of DAGitty. net to formalise causal relationships. Specific data points, such as accounts of trust‐building, variation in reviewer‐reviewee dynamics, and organisational responses to feedback, were mapped onto relational nodes and directional pathways, allowing us to visualise how interpersonal and contextual factors influence whether peer review results in compliance‐driven or learning‐oriented change.

In the context of peer review visits, a DAG helps to map out how different components, such as compliance assessments, shared learning, and trust‐building, interact to drive quality improvement. By distinguishing between direct effects (e.g., peer review visits leading to action plans) and mediating factors (e.g., staff engagement, leadership support), DAGs provide a more sophisticated understanding of how peer review mechanisms operate.

## 4 Results

We have structured the results section below in the following way. First, we will outline the key themes from the data originating with key stakeholder interviews. We report these themes narratively. We then grouped them through the lens of logic model domains. As is normal practice, we distinguish in the logic model between the categories of inputs, activities, outputs, outcomes and impact, as well as assumptions. Secondly, we describe the Theory of Change for peer review which we developed through the translation of the themes and logic model information. Thirdly, we present a visualisation of the theory of change model in the form of a DAG as it emerged from our empirical evidence. In a last step, we add to this visualisation the relational aspect of effective learning in health services which will demonstrate the shortcomings of constructing logic models and static theory of changes without accounting for the relational and trust building dimension of peer learning.

There were four key themes emerging from our key stakeholder interviews interpreted through the lens of the impact domain framework developed by Smithson et al. [34]. The first theme related to peer review as a compliance versus a learning mechanism. Respondents thought that there was a tension between the two objectives of the QNT organisational purpose which extended and defined also the practices and processes of peer review visits. Peer review visits were often perceived as a compliance tool rather than a learning process which at times, made services respond defensively to review visits and understand them as a regulatory activity rather than one to support learning.

… the assessment is made against compliance, against those which have been set down previously and some of which may or may not be entirely up to date and relevant… So, it's a little bit rigid in some senses that there are external factors that weren't taken into account and so I think because it's … you have to have fixed criteria to be assessed against but when those are out of date, the criteria, the system doesn't necessarily have a way of coping with that.

FLS 01

The second theme clearly emerging from our data analysis was that respondents understood the role of relationship building and trust development in peer reviews. Engagement levels and depths were seen as a function of the relational dynamics and, at times, linked to authoritative signalling which triggered surface level compliance but prevented deeper level learning in the visited organisation.

I don't think there is a process in place at the moment for how the individual services are going to learn unless there was a mechanism put in place by which there was suggested shared learning perhaps from the review of all of the services.

FLS 01

The short time duration of visits was also singled out as a barrier to effective relationship and rapport building reducing the potential for mutual learning. The lack of follow up and anticipations of lack of follow up also shaped perceptions of peer review visits as a compliance tool rather than as part of a process of ongoing interorganisational learning.

Thirdly, a theme was identified which captured the limited knowledge exchange and restricted shared learning of peer review visits, where respondents saw them as missed opportunities for knowledge sharing due to the fact that knowledge was often seen to flow from reviewers to services only. The one‐directional stream of information and expertise was mentioned repeatedly as a significant barrier to perceive of peer review visits as occasions of shared learning and mutual equitable exchange of views and experiences. A lack of systematic mechanism for cross organisational learning was seen as testament to the compliance and one‐sided nature of the knowledge exchange by respondents.

[The issue is] how do you feed this information into the wider intelligence about providers.

Workshop 02

The lack of a learning feedback loop was identified as a primary barrier to create genuine learning opportunities from peer review visits.

[What we would want to see is] almost reciprocal learning because you've got four or five centres and each is presumably going in and supporting the other, that is, you're right, I think you can sort of see more of a value.

Workshop participant 01

Lastly, respondents were unclear about how the existing processes of peer review visits could support quality improvement in the visited service. The long‐term impact of peer reviews was difficult to identify in the absence of a clear established feedback loop and follow up. It was difficult to understand, respondents argued, how a potential positive change was related to the inputs and mechanisms of the peer review visit to drive improvement in the long term. We took this to mean that there was a lack of a causal pathway from peer review activities to quality improvement.

I think the challenge… is around once you've done the peer review, how are you then going to make sure that those improvements are made… I'm not overly clear that we know what the next steps are. I think obviously there's feedback to the teams, and obviously it's gone to the chief execs and so on, and obviously internally we'll all be trying to make some improvements based on the report.

PR 01

### 4.1 Development of Theory of Change

Our analysis produced a comprehensive list of activities, inputs and outputs which are listed in the table below in the form of a logic model (Table 2).

**Table 2:** Logic model QNT peer review visits.

Category	Component	Description
Inputs	Human Resources	Multidisciplinary teams of peer reviewers, facilitators, and external experts
	Information and Data	Service documentation, self‐assessment data, patient pathway information
	Technological Resources	Digital tools for stakeholder engagement, data analysis, and reporting
	Time and Scheduling	Time allocated for participation, preparation, and implementation
	Funding and Logistics	Financial resources for travel, logistics, and facilitation
	Stakeholder Contributions	Engagement from commissioners, provider teams, and external stakeholders
	Knowledge and Expertise	Access to benchmarks, past peer review insights, and best practices
Activities	Preparation and Notification	Advance notification and self‐assessment preparation
	Engagement with Stakeholders	Interviews, discussions, and observational visits
	Compliance and Quality Assessments	Review of documentation and benchmarking against standards
	Facilitation of Shared Learning	Sharing best practices and collaborative learning discussions
	Feedback and Reporting	Draft and final reporting of findings and recommendations
	Action Planning and Monitoring	Development and monitoring of action plans
	Systemic Reflection and Adjustment	Identification of broader trends and systemic challenges
Outputs	Peer Review Reports	Draft and final reports outlining compliance, strengths, and recommendations
	Action Plans	Action plans detailing steps, responsibilities, and timelines
	Shared Learning Resources	Documents and sessions highlighting good practices and innovations
	Quality Benchmarks	Establishment of quality benchmarks and systemic issue identification
	Stakeholder Engagement Outcomes	Feedback incorporation and stakeholder engagement improvements
	Compliance Assessments	Assessment of compliance status and escalation needs
	Improvement Evidence	Tracking of progress and documented quality improvements
Outcomes	Short‐Term Outcomes	Awareness, engagement, and reflection on patient pathways and service delivery
	Medium‐Term Outcomes	Implementation of recommendations and evidence of practice improvement
	Long‐Term Outcomes	Tangible service improvements, systemic changes, sustainability, and accountability
Impact	Service‐Level Impacts	Enhanced quality, compliance, and pathway‐focused service improvements
	Staff and Organisational Impacts	Staff collaboration, learning culture, and professional development
	Patient‐Centred Impacts	Better patient experience, outcomes, and safety improvements
	System‐Wide Impacts	Standardisation, policy learning, and system‐wide accountability
	Challenges in Realising Impact	Sustainability of changes, balancing compliance versus quality improvement
Assumptions	Process Assumptions	Assumption of objectivity, credibility, and effective self‐assessment
	Impact Assumptions	Expectation of receptiveness to feedback and shared learning benefits
	Stakeholder Assumptions	Leadership support, patient‐centred improvements, and commissioner engagement
	Systemic Conditions Assumptions	Resource availability, systemic integration, and sustainability of changes
	Challenges to Assumptions	Potential resistance, misalignment with existing processes, and implementation challenges

The theory of change DAG illustration below represents a map of the contributing components and influencing factors to bring about change. In our DAG, to avoid overwhelming complexity and protect the benefits of visualisation, we did not differentiate between direct effects and excluded indirect or conditional effects.

**Figure 1:**
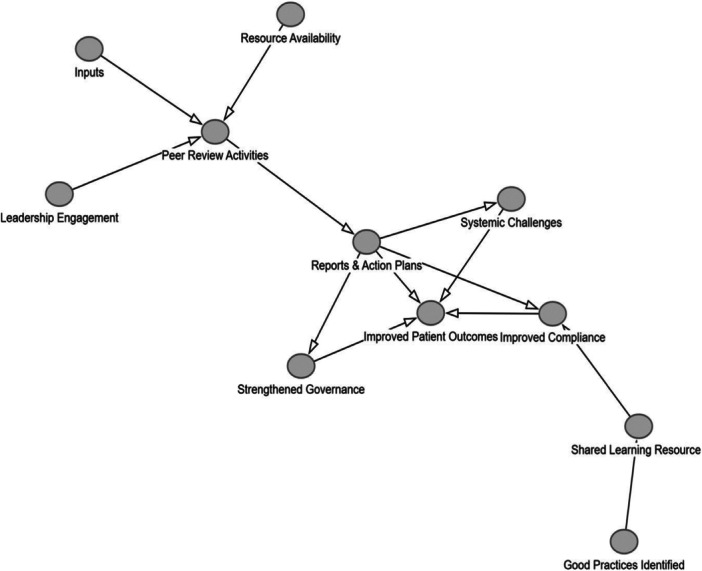
DAG peer review based on Logic model.

**Figure 2:**
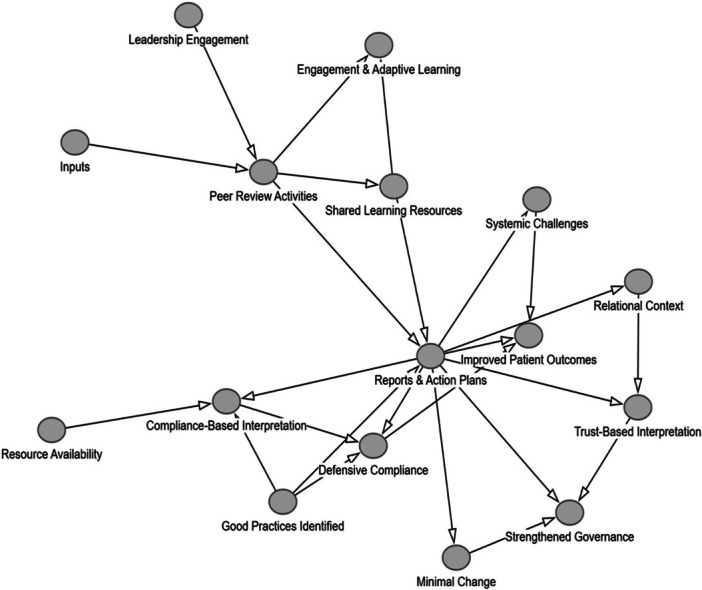
DAG peer review including relational dimension.

The first DAG (Figure 1) represents a static representation of the peer review process without taking into account the relationship and trust building factors as articulated by Etienne [33]. The second DAG further below (Figure 2), demonstrates the causal pathways of peer review practices when relationship and trust building impacts are included. We used the commonly used tool DAGitty. com to produce the graphs below.

The difference between the two figures demonstrates the gain and utility of creating DAGs when including dynamic elements of peer review processes as conceptualised by Etienne [33]. It also increases the explanatory power of the DAG which can be validated through future studies of peer review practice.

## 5 Discussion

The purpose of our study was to fill the conceptual gap in articulating a valid Theory of Change model for peer reviews in health care settings which permit researchers and practitioners to pinpoint active ingredients of peer review visits. This will support further investigation to identify pathways of impact and influencing factors in successful peer reviews. We used the analysis of an existing peer review visit to develop our theory of change and thus bridged the previously disconnected domains of empirical evidence and conceptual work in the field. Our Theory of Change model should allow investigators to articulate more clearly their approach to research and demonstrate the effect peer reviews have on organisations, staff as well as clinical units where appropriate. We will discuss our findings below in the context of existing evidence, followed by implications for practice and the theoretical contribution of our study.

Our study shows that peer review effectiveness is highly dependent on relational dynamics, an aspect which has been highlighted by Etienne's work [33]. Incorporating the relational dimension of peer reviews into our draft Theory of change moved it from a static to a dynamic model reflecting more closely the reality of practice and taking into account the interpersonal nature of some of the visits.

Etienne's paper pointed to the fact that the effectiveness of peer reviews is mediated by how it is perceived and enacted in relationships. This throws light at the limitations and disadvantages of the current model of peer review visits conducted by NHS England's QNT team but also provides generic insights into any other peer review practice in other health care settings. The dynamic nature of our finalised theory of change model also accommodates findings that indicated that peer reviews can lead to compliance‐driven or learning driven outcomes. This key finding of the dual nature and which factors swing peer review practice into which direction can now inform future studies and investigations of peer review practice. Our model moves away from predictable linear pathways assuming that peer review visits lead to recommendations and hence to service improvements. The more dynamic finalised version of our theory of change does not just clearly articulate key mechanisms and aligns them with expected outcomes but also accounts for the variability in how a peer review visit is perceived, received and enacted. It reconciles the informational with the behavioural dynamics and thus allows researchers to design more sophisticated and realistic study designs when investigating peer reviews with higher generalisability, a key requirement for high‐impact research.

### 5.1 Implications for Practice

There are several implications for practice emerging from our work. First of all, we propose to distinguish between compliance and quality improvement aspects of peer reviews. This recommendation is supported by previous research, albeit on insights stemming from mainly single site studies. At present, current NHS peer review processes blend compliance checks with learning objectives and it leads to the sending of mixed signals for providers. Within the restrictions of current policy, it may be useful to argue for a dual track system of peer review visits where learning‐based peer review operate separately from compliance and audit objectives. This relates to the second recommendation which suggests a shift from assessment model to developmental model. In conjunction with this recommendation we would advocate for longer review engagements and the establishment of a follow‐up mechanism. Our theory of change model indicates that short visits without follow‐up fail to build trust which is recognised in the literature as a precondition for strengthening the impact of peer reviews.

Moving from a static to a more dynamic understanding of peer review processes and in line with our recommendation to differentiate between compliance and learning processes, we point out that additional training of peer reviewers in relational signalling and trust building appears to be essential if learning effects are to be the primary focus of visits. Reviewers should be aware of the social and psychological dynamics of feedback to make sure their role is perceived as constructive rather than punitive.

Some work should also commence to understand how learning processes can be supported in visited organisations and which design changes have to be made to champion this dimension of peer review visits which aligns with the wider emerging learning health system approach advocated by NHS England.

### 5.2 Study Limitations

There are some limitations of our study. First, our findings are based on an analysis of the QNTs peer review visit practice, which imposes some limits on the generalisability of our empirical findings to all health care settings. However, the conceptual work we invested in the creation of the theory of change model represents a genuine advancement of hitherto mainly single‐site research with localised applicability and of limited validity. While the study employed independent coding and triangulation to enhance rigour, the qualitative nature of the analysis introduces the potential for researcher bias in theme interpretation and model construction. The modest sample size, although purposively diverse, limits generalisability, and the Theory of Change has not yet undergone external validation, which may constrain its applicability across different service contexts.

### 5.3 Future Research

We therefore suggest that our Theory of Change model should be validated empirically beyond our current localised verification through QNT staff and key stakeholders. In particular the systematic integration of knowledge exchange mechanisms in peer review processes is a primary practical recommendation emanating from our work. This also speaks to the function, purpose and sustainability of clinical specialty networks which often act as conveyor belts for knowledge exchange and intra‐specialty learning. The multidimensional character of peer reviews taking place on the organisational, team and individual level complicates the picture but we have demonstrated with our theory of change model that conceptual work can and must incorporate and reckon with the dynamic and multiplicity of potential effect and impact areas. It should also be noted that peer reviews being located within the interdisciplinary nature of healthcare work renders some of this work more challenging.

## 6 Conclusion

Our study advances the theoretical understanding of peer reviews by developing a theory of change that incorporates relational signalling and ambiguity. The revised theory of change model we present in our study is the first attempt to conceptualise impacts and effects of peer review visits in a systematic way which can be further explored in future research. Our work also utilises innovative theoretical modelling approaches which hopefully generates interest in the potential of future cross‐disciplinary efforts. In addition, our study puts ongoing investigations potentially on a more robust theoretical footing which will in time produce generalisable rigorous evidence on how to design and improve peer review practices as part of learning health systems where peer review processes are mediated by trust and learning engagement. We make a case to move beyond the compliance monitoring and focus instead on creating trust‐based, knowledge sharing learning environments.

## Ethics Statement

This service evaluation was conducted in accordance with the Health Research Authority (HRA) in the United Kingdom (UK) guidelines and, as a service evaluation, did not require formal ethical approval. The ethics committee of Edge Hill University deemed this study a service evaluation not requiring ethical review and approval.

## Conflicts of Interest

The authors declare no conflicts of interest.

## Supporting information

Appendix Verbatim Quotes.

## Data Availability

The data that support the findings of this study are available from the corresponding author upon reasonable request.
