# Supplementary material for: Beyond Compliance: Understanding the Role of Peer Review Through a Theory of Change
Source: J Eval Clin Pract. 2026 Mar 26;32(2):e70422. doi: 10.1111/jep.70422 (PMC13021267; doi:10.1111/jep.70422)
Supplement: Supplementary file 1 — Appendix Verbatim Quotes. [file JEP-32-0-s001.docx]

**Appendix Verbatim Quotes**

One key quote is provided below for each of the eight domains of the Impact Framework which guided the deductive analysis.

Anticipatory

“… the assessment is made against compliance, against those which have been set down previously and some of which may or may not be entirely up to date and relevant… So it’s a little bit rigid in some senses that there are external factors that weren’t taken into account and so I think because it’s … you have to have fixed criteria to be assessed against but when those are out of date, the criteria, the system doesn’t necessarily have a way of coping with that” (FLS 01).

Directive

“I think the challenge… is around once you’ve done the peer review, how are you then going to make sure that those improvements are made… I’m not overly clear that we know what the next steps are. I think obviously there’s feedback to the teams, and obviously it’s gone to the chief execs and so on, and obviously internally we’ll all be trying to make some improvements based on the report. But I think for our particular service, I don’t think we know what the next steps are. Whereas normally with peer reviews and quality improvement programmes, you’re having a period of time to make that improvement, and then you’ll know when the next review’s gonna take place. I think with the peer review, I don’t think any of us know when the next one would be. So, it leaves it a little bit open ended as to whether you feel it’s a quality improvement or not, because you could have feedback and then not do anything with the feedback” (PR 01).

Organisational

“We have managed to tick all those things off and we have got the paperwork for that but if they told me there were coming tomorrow I would be like, oh my god, not everything is in the right folder, in the right place, I’m going to have to sit and sort all of that out again but its more than a four lined email now, it is a SOP and pathways and those essential things but it hasn’t really changed anything in the way we deliver our service, its literally just writing down stuff on a bit of paper…” (QNT 04).

Relational

“It certainly did feedback on some occasions that there need to be change so it wasn’t soft in circumstances, but it didn’t provide expertise in how to do it. It just said this needs to be done for your service kind of thing” (QNT 05).

“Yes, so you've got somebody who, you know… has to transport patients for four or five hours, where that's not something that, you know, [city] would ever do, and were very critical of things that were out of the trust's control because of the geography” (COM 01).

Informational

“I think there is nowhere for comparison for trusts to see each other’s reports so once that final report goes back to the organisation then it’s not seen, it’s not held publicly anywhere or anywhere within NHS England where other people can actually dip in and see them and I feel that they’re useful for other members even within the team” (QNT 03).

Stakeholder

“I think it’s very crucial to include patient perspectives in the reviews and I think we are missing that because we do have patients [on the review team] that come in but don’t have a relationship with the staff who work in the service. It is more important to hear from the patients who are actually using the service” (QNT 06).

Lateral

“So I don’t think there’s been and I’m not aware at the moment of a process by which for example the reports from the different centres are shared or discussed in terms of looking at them together… I don’t think there is a process in place at the moment for how the individual services are going to learn unless there was a mechanism put in place by which there was suggested shared learning perhaps from the review of all of the services” (FLS 01).

“[What we would want to see is] almost reciprocal learning because you’ve got four or five centres and each is presumably going in and supporting the other, that is, you’re right, I think you can sort of see more of a value” (Workshop participant 01)

Systemic

“… the way we work with CQC is they do use our information. CQC like to have more broader hospital information and they would like us to have a more of a traffic light system in terms of where we thought somebody was good, bad or indifferent or whatever we want to say… So that doesn't always lend itself to what peer review use, but they certainly get our reports they asked for a breakdown every year of certain services and we provide it to them. So we do work with them” (QNT 02).

“[The issue is] how do you feed this information into the wider intelligence about providers. You cannot have alarm bells ringing in one part of the system if actually some of that issue is around governance or leadership, because that has implication on all services, even if specialised commissioning, don't pay the bill.” (workshop 02)
